# 

**DOI:** 10.1055/s-0035-1570109

**Published:** 2016-01

**Authors:** Alina Coutinho Rodrigues Feitosa, Amado Nizarala de Ávila

**Affiliations:** 1 Escola Bahiana de Medicina e Saúde Pública, Salvador, BA, Brasil; 2 Serviço de Endocrinologia da Maternidade Prof José Maria de Magalhães Netto, Salvador, BA, Brasil; 3 Residência de Obstetrícia da Maternidade Prof José Maria de Magalhães Netto, Salvador, BA, Brasil

**Keywords:** diabetes mellitus, gravidez em diabéticas, diabetes gestacional, sistemas computadorizados de registros médicos, doenças do sistema endócrino, diabetes mellitus, pregnancy in diabetics, gestational diabetes, medical records systems, computerized, endocrine system diseases

## Abstract

**Objetivo** Apresentar e validar um registro eletrônico de saúde (RES) multifuncional para atendimento ambulatorial a portadoras de endocrinopatias na gestação e comparar a taxa de preenchimento de informações de saúde com o prontuário convencional.

**Métodos** Desenvolvemos um RES denominado Ambulatório de Endocrinopatias na Gestação eletrônico (AMBEG) para registro sistematizado das informações de saúde. O AMBEG foi utilizado para atendimento obstétrico e endocrinológico de gestantes acompanhadas no ambulatório de endocrinopatias na gestação na maternidade referência em gestação de alto risco na Bahia, no período de janeiro de 2010 a dezembro de 2013. Aleatoriamente foram selecionadas 100 pacientes atendidas com o AMBEG e 100 pacientes atendidas com prontuário convencional com registro em papel e comparou-se a taxa de preenchimento de informações clínicas.

**Resultados** Foram realizados 1461 atendimentos com o AMBEG: 253, 963 e 245 respectivamente, admissões, consultas de seguimento e puerpério. Eram portadoras de diabetes 77,2% e sendo 60,1% portadoras de diabetes pré-gestacional. O AMBEG substituiu, satisfatoriamente, o prontuário convencional. O percentual de informações clínicas registradas em ambos os prontuários foi significativamente maior no AMBEG: queixas clínicas (100 *versus* 87%, *p* < 0,01), altura uterina (89 *versus* 75%, *p* = 0,01), ganho de peso total (91 *versus* 40%, *p* < 0,01) e dados específicos sobre o diabetes (dieta, esquema de insulina, controle glicêmico e manejo de hipoglicemias) revelando diferença significativa ( *p* < 0,01). A possibilidade de exportar dados clínicos para planilhas facilitou e agilizou a análise estatística de dados.

**Conclusões** O AMBEG é uma ferramenta útil no atendimento clínico a mulheres portadoras de endocrinopatias na gestação. A taxa de preenchimento de informações clínicas foi superior à do prontuário convencional.

## Abstract (en)

**Objective** To present and validate a multifunctional electronic medical record (EMR) for outpatient care to women with endocrine disorders in pregnancy and to compare health information data fill rate to conventional medical records.

**Methods** We developed an EMR named Ambulatory of Endocrine Diseases in Pregnancy (AMBEG) for systematic registration of health information The AMBEG was used for obstetric and endocrine care in a sample of pregnant women admitted to the maternity reference in high-risk pregnancies in Bahia from January 2010 to December 2013. We randomly selected 100 patients accompanied with AMBEG and 100 patients monitored with conventional consultation and compared the health information data fill rate of the electronic consultation to that performed using conventional medical records.

**Results** 1461 consultations were held, of which 253, 963 and 245 were first, follow-up and puerperium consultations, respectively. Most patients were pregnant women with diabetes (77.2%) and 60.1% were women with pre-gestational diabetes. The AMBEG satisfactorily replaced the conventional medical record. The percentage of registered information was significantly higher in the AMBEG: clinical symptoms (87% versus 100, *p* < 0.01), uterine height (89 versus 75%, *p* = 0.01), total weight gain (91 versus 40%, *p* < 0.01) and specific diabetes data (diet, insulin regimen, glycemic control and management of hypoglycemia) revealed a significant difference ( *p* < 0.01). The ability to export data to worksheets greatly facilitated and accelerated the statistical analysis of the data.

**Conclusions** AMBEG is a useful tool in clinical care for women with endocrine diseases during pregnancy. The fill rate of clinical information was superior to that registered in conventional medical records.

## Introdução

A presença de diabetes mellitus na gestação está associada a elevado risco de complicações. 1 É uma condição que consome recursos de saúde 2 e atenção especializada. A prevalência do diabetes pré-gestacional e do gestacional tem aumentando nos últimos anos e pode ser justificada pela epidemia de obesidade 3 4 , aumento da idade materna 4 e pelo recente e sensível critério para diagnóstico do diabetes gestacional. 5 A fim de reduzir a chance de maus desfechos, gestantes portadoras de diabetes devem ter assegurada a sua assistência pré-natal em centros de referência especializados e, preferencialmente, terem acompanhamento multidisciplinar. Intervenções dietéticas, mudanças no estilo de vida, monitoração das glicemias, manutenção dos alvos glicêmicos rigorosos, pré-natal com vigilância específica e controle do peso são indispensáveis para otimizar as metas de tratamento e os desfechos materno-fetais 6 , entretanto exigem, do médico e da equipe de saúde, sistematização do atendimento, seguimento das diretrizes e rigor na avaliação e conduta.

Tecnologias de informação e comunicação em saúde (TICS) tem o potencial de otimizar a eficiência e efetividade dos profissionais de saúde. 7 Dentre as TICS, o prontuário eletrônico do paciente (PEP) é a principal ferramenta. Outro recurso utilíssimo, embora com menos funcionalidades, é o registro eletrônico de saúde (RES), que é um repositório de informação a respeito da saúde de indivíduos, processável eletronicamente e que permite armazenamento e compartilhamento de informações. No RES a informação é mais disponível e atualizada, os dados têm maior legibilidade, acurácia e exatidão e, por meio de sistemas de alerta e apoio à decisão terapêutica associados ao RES, a possibilidade de erro é potencialmente reduzida, aumentando a segurança para o paciente. 7

O uso do RES em portadores de diabetes está associado a melhores taxas de intensificação de tratamento, monitoração, seguimento e otimiza o controle glicêmico e lipídico. 8 O PEP e o RES, tornam-se então, hipoteticamente, ferramentas atraentes para o atendimento da gestante portadora de diabetes onde a otimização clínica, com objetivo de normoglicemia, assegura melhor desfecho materno-fetal. Entretanto não há, ao nosso conhecimento, RES brasileiro validado para atendimento desta população específica.

O objetivo do presente estudo é apresentar um registro eletrônico de saúde desenvolvido para atendimento de portadoras de endocrinopatias na gestação, com ênfase em diabetes, e os resultados do acompanhamento de gestantes no período de três anos, comparando a taxa de preenchimento das informações do prontuário eletrônico ao convencional.

## Métodos

O presente trabalho reporta o desenvolvimento e a validação de um aplicativo de banco de dados para atendimento de gestantes portadoras de diabetes no pré-natal e um estudo do tipo caso-controle para comparar o atendimento médico por meio do prontuário eletrônico ao convencional.

Foi desenvolvido um aplicativo do banco de dados do Microsoft Access®, denominado Ambulatório de Endocrinopatias na Gestação eletrônico (AMBEG), para o registro eletrônico sistematizado de informações de saúde para o atendimento médico obstétrico e endocrinológico. Após o desenvolvimento e teste, o AMBEG foi utilizado para o atendimento clínico de uma amostra populacional consecutiva de pacientes acompanhadas ao ambulatório de endocrinopatias na gestação da Maternidade Professor José Maria de Magalhães Netto (MPJMMN) durante o período de janeiro de 2010 a dezembro de 2013. A pesquisa foi aprovada pelo comitê de ética em pesquisa local.

Para o desenvolvimento do AMBEG utilizou-se o aplicativo Microsoft Access®, que é um sistema de gerenciamento de banco de dados da Microsoft que permite o desenvolvimento rápido de aplicações que envolvem modelagem, estrutura de dados e interface a ser utilizada pelos usuários. É capaz de utilizar dados armazenados em *access/Jet, Microsoft SQL Server, Oracle* e quaisquer dados compatíveis com *Open Database Connectivity* . Os campos de dados, formato de distribuição, construção dos formulários foram criados a partir da organização habitual dos prontuários no atendimento convencional, sequenciando a ordem de entradas dos campos de acordo com a entrevista médica. Com o foco no acompanhamento do diabetes, foram acrescidos campos que garantissem a entrada de dados sobre todos os aspectos relevantes do acompanhamento clínico como: dieta, tratamento, automonitoração, uso de insulinas e hipoglicemias. Foram criadas telas para atendimento à primeira consulta, acompanhamento evolutivo da paciente (“retorno”), registro de dados laboratoriais, ultrassonografia obstétrica e dados de puerpério da portadora de patologias endocrinológicas na gestação. O AMBEG seguiu as orientações e inclui todos os itens obrigatórios do prontuário eletrônico definidos pela resolução do CFM n. 1.638/2002. 9

Os usuários do AMBEG foram o médico endocrinologista e os médicos residentes de obstetrícia e endocrinologia que cumpriam estágio regular no ambulatório de endocrinopatias na gestação. Os médicos usuários eram ensinados a utilizar o AMBEG através de demonstração breve de cerca de dez minutos e treinados durante um atendimento. O aplicativo é autoexplicativo e tem campos com mensagens, caixas de listas e máscaras que controlam a entrada de dados. Ao final da consulta, o atendimento era impresso, assinado e arquivado.

A avaliação do desempenho do aplicativo foi feita por meio da comparação da taxa de preenchimento de dados do atendimento no prontuário preenchido a mão, denominado “atendimento convencional“ com a do preenchimento no prontuário eletrônico, denominado “atendimento eletrônico”. Consideramos a diferença da taxa de preenchimento do dado “queixas clínicas” como o desfecho principal para o cálculo do tamanho amostral. Estimando-se uma diferença média na taxa de preenchimento de 15%, com o poder de 80% e o nível de significância de 5%, o tamanho da amostra calculado para cada grupo foi de 99 indivíduos. Por meio de números aleatórios gerados em Microsoft Excel® foram selecionados 100 prontuários preenchidos por atendimento convencional e 100, por atendimento eletrônico.

As informações registradas no atendimento do primeiro retorno foram utilizadas para comparar a taxa de preenchimento de informações entre os dois tipos de atendimento. Foram escolhidas variáveis consideradas pelos autores como essenciais e representativas do atendimento à portadora de diabetes na gestação e categorizados em nove domínios de dados: 1. Dados sobre queixas: descrição de presença ou negação de queixas clínicas ou obstétricas; 2. Datação da gestação: descrição da idade gestacional; 3. Exame físico: descrição da pressão arterial, altura do fundo uterino, peso materno e ganho total de peso; 4. Aspectos nutricionais: descrição do uso de adoçante artificial e descrição de aderência ou não à dieta; 5. Uso de medicações: descrição de hipoglicemiantes orais e de suplementos maternos; 6. Dados sobre insulinoterapia: informação sobre o uso de insulina e esquema de insulinização; 7. Dados de automonitoração: registro de percentuais de glicemia acima, dentro e fora da meta; 8. Dados sobre hipoglicemias: descrição sobre história de hipoglicemia no período interconsulta e sobre reconhecimento e tratamento de hipoglicemias; descrição sobre o uso ou não do cartão do diabético; 9. Dados sobre conduta: informações sobre alteração ou não da conduta. Era considerada informação “presente” quando o campo estava preenchido e “ausente” quando não houvesse informações.

Todos os dados inseridos nos campos da entrevista médica eram, por comando simples, exportados para planilhas de Excel, que possibilitavam a análise estatística.

A análise estatística foi feita com programa SPSS v. 20.0 para Windows ^TM^ . Os dados dos registros eletrônicos foram expressos como média e desvio-padrão ou mediana e variação e as variáveis categóricas, como valor absoluto e percentual. Variáveis com dados dicotômicos foram comparadas utilizando-se o teste do qui-quadrado ou pelo exato de Fisher.

## Resultados

**Fig. 1:**
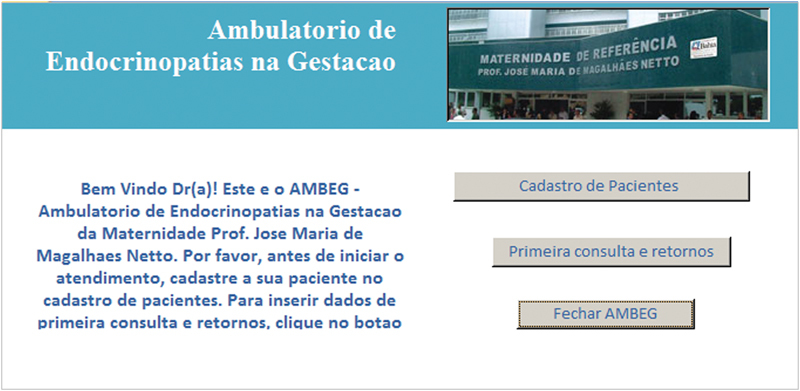
Tela inicial.

O AMBEG foi desenvolvido com uma tela primária, para direcionamento da navegação por meio de botões de cadastro de egressas, acesso à primeira consulta e retornos e o fechamento da ferramenta ( Figs. 1 2 3 4 5 6 7 8 ).

**Fig. 2:**
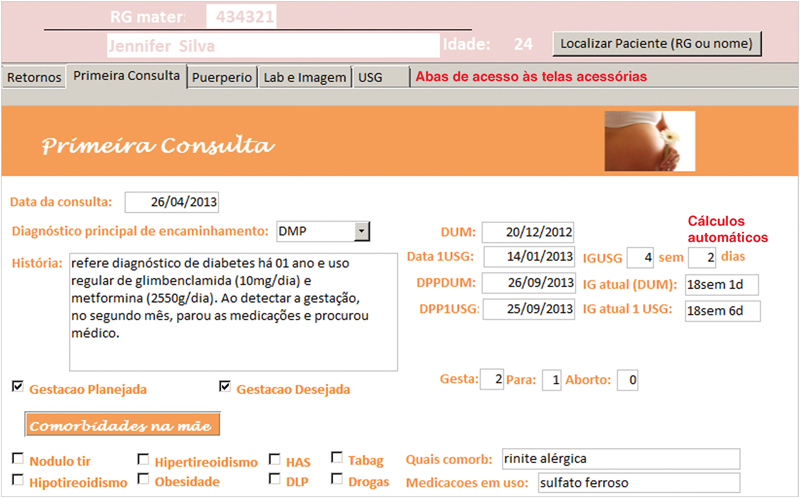
Tela de primeira consulta (demonstração parcial).

**Fig. 3:**
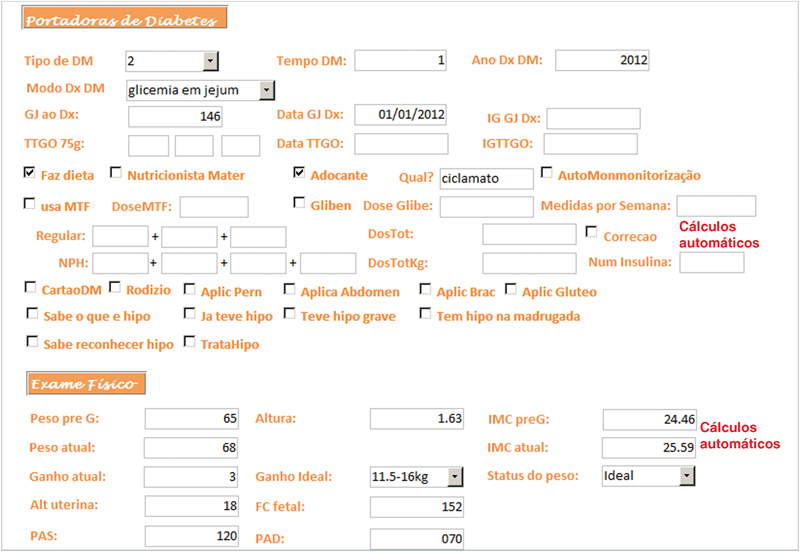
Tela de primeira consulta, parte específica para as diabéticas (demonstração parcial).

**Fig. 4:**
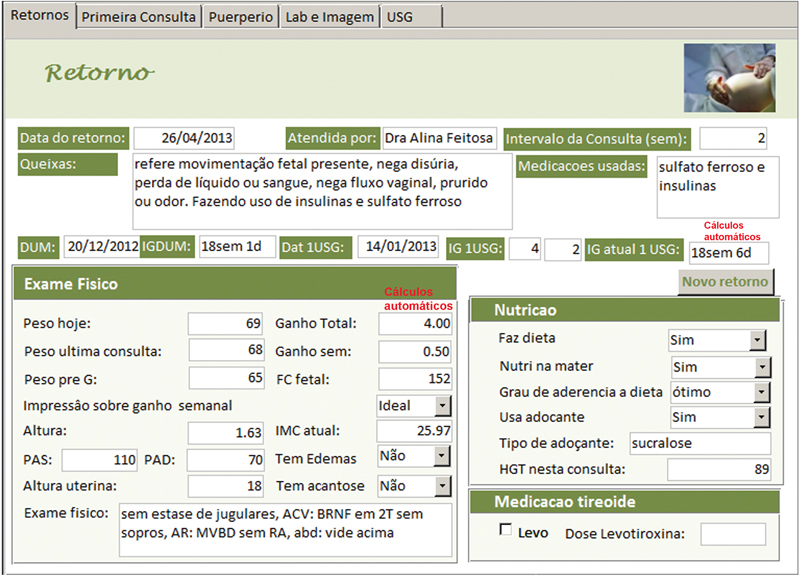
(a) Tela de retorno à consulta (demonstração parcial). (b) Tela de retorno à consulta, avaliação específica da diabética gestante, incluindo a conduta (demonstração parcial).

**Fig. 5:**
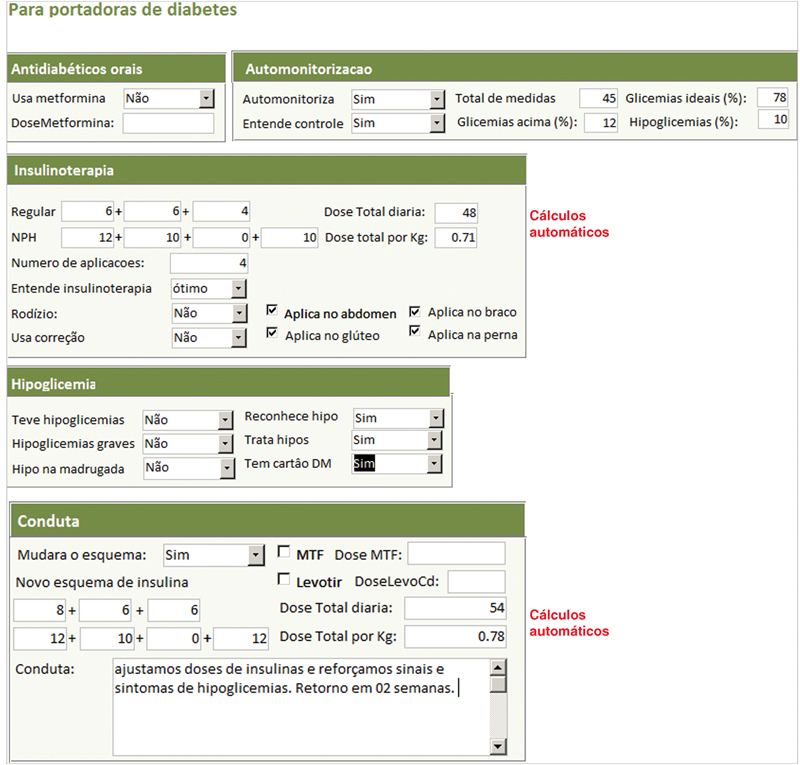
Tela de retorno à consulta – avaliação específica para diabetes.

**Fig. 6:**
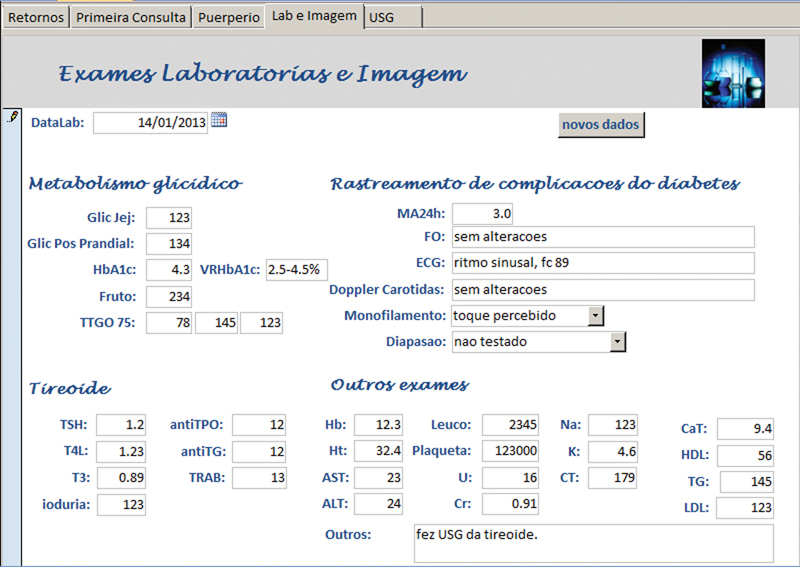
Tela para exames laboratoriais e de imagem (tireoide).

**Fig. 7:**
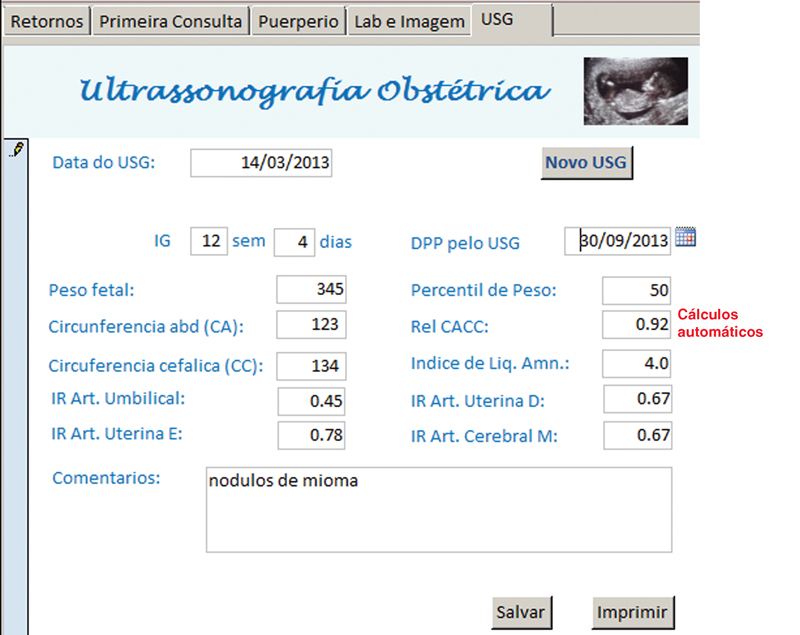
Tela para exame de ultrassonografia obstétrica.

**Fig. 8:**
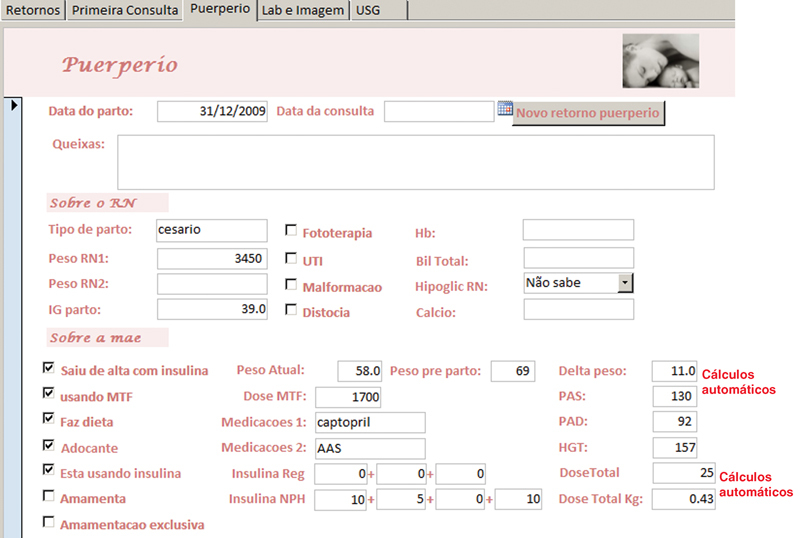
Tela para atendimento no puerpério (demonstração parcial).

A anamnese completa e exame físico foram registrados nos campos da tela “primeira consulta” e as abas na borda superior davam acesso às telas de “retorno”, “puerpério”, “lab e imagem” e “USG”, onde eram inseridos, a cada consulta, respectivamente, dados sobre a evolução durante a gravidez, do puerpério, exames laboratoriais, de imagem e ultrassonografia obstétrica. Em todas as telas de atendimento existiram campos alfa numéricos, que permitem escrita livre; campos numéricos, para variáveis contínuas como peso, altura, pressão arterial, altura do fundo uterino, batimentos fetais, idade gestacional, etc.; campos apoiados por listas permitem entradas categóricas predeterminadas e campos numéricos com cálculos automáticos, para otimizar e agilizar o atendimento como o cálculo da idade gestacional (IG) e data prevista do parto pela data da última menstruação (DUM) e a primeira ultrassonografia; ganho de peso total e semanal, índice de massa corpórea (IMC), dose total diária (DTD) de insulina e dose por quilo de peso, intervalo de tempo entre consultas, etc.

Os usuários do AMBEG foram os médicos endocrinologistas e residentes de endocrinologia e obstetrícia, sendo que, estes últimos, faziam estágio regular no ambulatório de endocrinopatias na gestação por período de dois meses. A maioria dos médicos que utilizaram o AMBEG relataram estar muito satisfeitos com o uso e a impressão foi que o AMBEG era de fácil manejo, capaz de organizar a sequência de atendimento e minimizar falhas. Os cálculos automáticos no AMBEG auxiliaram e agilizaram o atendimento, segundo os usuários (dados não mostrados).

Durante o período de utilização, a necessidade de suporte técnico foi mínima e não ocasionou nenhum prejuízo ao atendimento.

Por meio do aplicativo eletrônico 319 pacientes foram atendidas. Considerando-se primeiras consultas e consultas de retorno e puerpério, um total de 1460 atendimentos foram realizados no período avaliado, sendo 252 primeiras consultas, 963 “retornos” e 245 avaliações em puerpério. Sessenta e sete pacientes foram registradas pela primeira vez na tela “Retorno”, mas eram primeira consulta. Haviam sido admitidas e feita a primeira consulta na internação, em prontuário preenchido a mão.

Dados sobre a primeira consulta estão sumarizados na Tabela 1 ( *n* = 246). A média de idade materna foi de 32 ± 10 anos e idade gestacional de 25 ± 7,8 semanas. O principal motivo de encaminhamento ao endocrinologista foi o diabetes (77,2%), seguido de patologias tireoidianas. O diabetes pré-gestacional foi o principal tipo de diabetes encaminhado correspondendo a 60,1% dos casos ( Tabela 1 ).

**Tabela 1:** Dados clínicos das gestantes na primeira consulta (expressos em média e desvio-padrão, valor absoluto e percentual)

Variáveis	Todas ( *n* = 252) ** n = 246	Somente DM ( *n* = 193) ** n = 183
Idade (anos)	32 ± 10	32 ± 10
IG (semanas)	25 ± 7,8	25,5 ± 7,9
Diagnóstico ao encaminhamento *	DMP *n* = 107 (43,5%); DMG *n* = 83 (33,7%); Tir *n* = 45 (18,3%); Obes *n* = 3 (1,2%); Hip *n* = 1 (0,4%); Outros† *n* = 7 (2,9%)	DM1 *n* = 25 (13,7%) DM2 *n* = 65 (46,4%%) DMG *n* = 73 (39,9%)
Peso pré-gestacional (kg)	72,4 ± 16,8	73,6 ± 16,7
IMC pré-gestacional (kg/m ^2^ )	28,5 ± 6,1	28,9 ± 6,0
Peso na PC (kg)	79,8 ± 16,8	81 ± 16,3
Ganho de peso até PC (kg)	7,7 ± 7,9	7,5 ± 6,7
Glicemia capilar na PC (mg/dl)	138 ± 65,5	146 ± 68,5

Abreviações: DM, diabetes mellitus; DM1 diabetes mellitus tipo 1; DM2 diabetes mellitus tipo 2; DMG diabetes gestacional; DMP diabetes mellitus pré-gestacional; Hip doenças hipotálamo hipofisárias; IG, idade gestacional; IMC, índice de massa corpórea; Obes, obesidade; PC, primeira consulta; Tir, doenças tireoidianas; †outras patologias endocrinológicas

* patologia que motivou o encaminhamento ao ambulatório de endocrinopatias na gestação.

** no grupo *Todas* , *n* = 6 estavam sem a identificação da patologia à primeira consulta; no grupo *Somente DM* , em 10 não havia a definição do tipo de diabetes.

A comparação da frequência de registro de informações nos nove domínios de dados entre os dois tipos de atendimento está demonstrada na Tabela 2 . A frequência do registro de dados foi significativamente maior no atendimento por prontuário eletrônico que pelo convencional nos campos de Queixas (100 *versus* 87%, *p* < 0,01), altura do fundo uterino (89 *versus* 75%, *p* = 0,01), ganho total de peso (91 *versus* 40%, *p* < 0,01) e questões especificas para as portadoras de diabetes: dieta (81,5 *versus* 8,4%, *p* < 0,01), esquema de insulina (70,4 *versus* 41%, *p* < 0,01), controle glicêmico (63 *versus* 15,7%, *p* < 0,01), história de hipoglicemias (72,8 *versus* 33,7%, *p* < 0,01), reconhecimento (72,8 *versus* 33,7%, *p* < 0,01) e tratamento de hipoglicemias (72,8 *versus* 33,7%, *p* < 0,01) e se porta consigo o cartão de diabetes (72,8 *versus* 30,1%, *p* < 0,01).

**Tabela 2:** Frequência de preenchimento dos dados clínicos conforme tipo de prontuário ( *n* = 200)

Variável	P. eletrônico ( *n* = 100)	P. convencional ( *n* = 100)	
	%	%	*p*
Queixas	100	87	< 0,01
Datação da gestação	91	83	0,09
Pressão arterial	93	92	0,79
Altura do fundo uterino	89	75	0,01
Peso materno	96	92	0,23
Ganho total de peso	91	40	< 0,01
Glicemia capilar na consulta	80	77	0,61
Conduta	95	97	0,47
Portadoras de diabetes mellitus ( *n* = 164)	% ( *n* = 81)	% ( *n* = 83)	*p*
Adoçante	81,5	63,9	0,01
Dieta para diabetes	81,5	8,4	< 0,01
Hipoglicemiantes orais	84	73,5	0,10
Uso de insulina	88,9	80,7	0,15
Esquema de insulina	70,4	41	< 0,01
Controle glicêmico (percentuais dentro do alvo)	63	15,7	< 0,01
História de hipoglicemias	72,8	33,7	< 0,01
Reconhece hipoglicemias	72,8	33,7	< 0,01
Sabe tratar hipoglicemias	72,8	33,7	< 0,01
Tem o cartão da diabética?	72,8	30,1	< 0,01

## Discussão

O uso da informação clínica eletrônica tem o potencial de melhorar a qualidade e a eficiência do cuidado médico. 10 11 No presente trabalho apresentamos um RES, o AMBEG, que foi desenvolvido para o atendimento a portadoras de endocrinopatias na gestação, com ênfase em diabetes. Neste trabalho também relatamos a experiência com três anos de assistência clínica endocrinológica utilizando o AMBEG e o desempenho do novo aplicativo comparado ao prontuário convencional.

Nos últimos anos tem havido especial interesse e investimento em tecnologias de informação e comunicação em saúde em várias partes do mundo. 10 No Brasil, o PEP/RES vem sendo utilizado com crescente frequência e resolução do CFM regulamenta o seu uso. 7 Evidências se avolumam sobre a importância dos PEP nos cuidados clínicos 10 11 e melhoria da qualidade da assistência ambulatorial. 12 em diabetes, o uso de prontuários eletrônicos tem o potencial de contribuir nos cuidados e prevenção de morbidades, 13 auxiliando na solicitação de exames de rastreamento 14 e recomendações específicas sobre medicações como insulinas. Na assistência ao diabetes fora do período gestacional, o uso do PEP completo e certificado está associado a melhoria nas taxas de intensificação do tratamento, seguimento, monitoramento, controle lipídico e glicêmico, 8 a menos visitas às emergências e internações. 15 Entretanto poucas pesquisas tem sido conduzidas sobre registros eletrônicos de saúde no campo dos cuidados pré- e perinatais na portadora de diabetes 16 e desconhecemos publicações brasileiras sobre o assunto. No presente estudo um RES foi criado e testado para assistência específica à portadora de doença endócrina na gestação com ênfase em diabetes, minimizando esta lacuna de conhecimento. Os dados obtidos dos atendimentos por meio do prontuário eletrônico demonstraram que a principal doença motivadora do encaminhamento obstétrico ao endocrinologista foi o diabetes, refletindo a frequência, impacto da doença na gestação e a procura por serviço de atendimento especializado. A população avaliada demonstrou ser de elevado risco pois a maioria eram portadoras de diabetes prévio à gestação.

Os médicos que utilizaram o AMBEG aprenderam rapidamente como operar o RES. Relataram estar satisfeitos com as funcionalidades, referiram ser de fácil manejo e capaz de organizar a sequência de atendimento, minimizando falhas. Reportaram também que os cálculos automáticos foram úteis e agilizaram o atendimento. Ainda que ginecologistas e obstetras revelem elevada satisfação com os sistemas eletrônicos para atendimento ao paciente 17 , idade mais avançada do médico, sexo masculino e atendimento clínico solitário foram barreiras para a implementação de prontuários eletrônicos. O RES do presente estudo teve elevada aceitação e fácil implementação possivelmente por serem os usuários médicos com pouco tempo de formação.

O presente trabalho também demonstrou a facilidade no acesso às informações coletadas, armazenamento e análise dos dados clínicos registrados eletronicamente. O registro clínico em prontuários preenchido a mão impõe dificuldades no acesso e levantamento de dados, limitando avaliações de qualidade de atendimento, consultas e pesquisas médicas. O registro eletrônico no AMBEG possibilitou a exportação rápida de informações com a criação de bancos de dados. Os campos estruturados permitiram consulta rápida e análise, fornecendo informações para pesquisas e levantamentos epidemiológicos. A utilização de dados de prontuários eletrônicos para dar suporte a pesquisas clínicas é uma tendência mundial e iniciativas, como o projeto *Eletronic Health Records for Clinical Research* 18 , procuram desenvolver inventário de dados necessários para dar suporte a pesquisas por meio de PEP. Nosso estudo demonstra a utilidade do RES para pesquisas e levantamentos epidemiológicos.

A frequência do registro de dados clínicos considerados relevantes na consulta foi significativamente maior no atendimento feito com o AMBEG em relação ao atendimento convencional, em papel. A estruturação dos campos de dados de consulta, a legibilidade, praticidade e os cálculos automáticos provavelmente auxiliaram na organização do atendimento e na lembrança do questionamento dos dados da consulta. Estas funcionalidades e os resultados do presente estudo segue a direção atual dos conhecimentos que demonstram a utilidade do registro eletrônico em melhorar a qualidade do registro clínico. 19 Em contrapartida a falta de documentação de informações do pré-natal resulta em impacto negativo nos escores de adequação dos cuidados pré-natais 20 e possivelmente o fato de haver melhor documentação de dados poderia resultar em melhoria na qualidade de assistência.

Apresentaram-se como limitações do trabalho, o tipo do PEP e a ausência de avaliação dos desfechos materno-fetais. O AMBEG é um PEP básico, que funciona como um registro eletrônico de saúde. Os PEP completos 21 oferecem outras funcionalidades consideradas importantes para o manejo clínico. 22 Entretanto, o AMBEG é um aplicativo transitório e em evolução. A ele poderão ser, posteriormente, integradas funcionalidades avançadas como a interoperacionalidade com outros sistemas (laboratório, imagem, prontuários de outras unidades), inserção de ordem de cuidados médicos eletrônica (prescrição, requisição de exames), incorporação de *links* com informações médicas para educação continuada e sistemas de alertas e avisos que funcionam como apoio a decisão diagnóstica e terapêutica. Adicionalmente, o registro de informações foi significativamente mais frequente na consulta realizado com o AMBEG, entretanto não avaliamos o impacto do uso na qualidade da assistência pré-natal e perinatal da gestante portadora de diabetes. Acreditamos que a sistematização do atendimento e o preenchimento mais completo dos dados relevantes da consulta possam se associar a redução de eventos maternos e fetais, o que poderá ser investigado em outros estudos. A informação sobre o tempo de atendimento não foi registrada para permitir a comparação, portanto não podemos assegurar se o prontuário eletrônico reduz o tempo de consulta, mas esta funcionalidade também pode ser agregada ao aplicativo possibilitando avaliações futuras.

Como pontos fortes do trabalho destacamos a originalidade do RES e ausência de publicações sobre o assunto na literatura brasileira. Diante das dificuldades de assistência em saúde no Brasil, da crescente prevalência do diabetes na gestação com suas complicações e da necessidade de padronização de atendimento e da conduta para otimizar as metas em saúde, demonstramos a utilização de uma ferramenta de baixo custo com suporte técnico mínimo capaz de aumentar a frequência do registro de dados clínicos no pré-natal de portadoras de diabetes na gestação. A maior frequência de registro de dados clínicos sobre hipoglicemias merece destaque pois estas informações representam atenção dedicada à segurança para a binômio materno-fetal o que poderia reduzir morbidades. O AMBEG tem o potencial de melhorar a assistência pré-natal de alto risco, incorporar tecnologias para otimizar os desfechos clínicos através da facilitação de aderência aos protocolos clínicos e assistenciais, oferecer mais segurança à paciente, além de proporcionar fonte de dados para levantamentos epidemiológicos e tratamento estatístico úteis para programas de atenção especializada ao diabetes na gestação e pesquisa clínica.

O registro eletrônico AMBEG é uma ferramenta de baixo custo, com necessidade de mínimo suporte técnico e fácil utilização que aumentou a frequência de registro de informações da consulta e padronizou o atendimento clínico a mulheres portadoras de endocrinopatias na gestação, com ênfase no diabetes. O potencial de expansão da ferramenta é amplo com perspectiva de melhora no atendimento por meio de tecnologia incorporada como alertas e interoperacionalidade com outros sistemas ambulatoriais e hospitalares. Recomenda-se mais estudos para avaliar o impacto a utilização da ferramenta nos desfechos clínicos.

## References

[1] International Association of Diabetes and Pregnancy Study Groups Consensus Panel Metzger B E Gabbe S G Persson B International association of diabetes and pregnancy study groups recommendations on the diagnosis and classification of hyperglycemia in pregnancy Diabetes Care 2010 33 03 676 682 20190296 10.2337/dc09-1848 PMC2827530

[2] Law A McCoy M Lynen R The prevalence of complications and healthcare costs during pregnancy J Med Econ 2015 18 07 533 541 25714263 10.3111/13696998.2015.1016229

[3] McIntyre H D Gibbons K S Flenady V J Callaway L K Overweight and obesity in Australian mothers: epidemic or endemic? Med J Aust 2012 196 03 184 188 22339524 10.5694/mja11.11120

[4] Morikawa M Yamada T Yamada T Sato S Cho K Minakami H Prevalence of hyperglycemia during pregnancy according to maternal age and pre-pregnancy body mass index in Japan, 2007-2009 Int J Gynaecol Obstet 2012 118 03 198 201 22727054 10.1016/j.ijgo.2012.04.019

[5] Mayo K Melamed N Vandenberghe H Berger H The impact of adoption of the international association of diabetes in pregnancy study group criteria for the screening and diagnosis of gestational diabetes Am J Obstet Gynecol 2015 212 02 2240 2.24E11 10.1016/j.ajog.2014.08.027 25173183

[6] American Diabetes Association (12) Management of diabetes in pregnancy Diabetes Care 2015 38(Suppl):S77 S79 25537713 10.2337/dc15-S015

[7] Conselho Federal de Medicina Cartilha sobre Prontuário Eletrônico: a certificação de sistemas de Registro Eletrônico de Saúde Brasília (DF) CFM 2012

[8] Reed M Huang J Graetz I Outpatient electronic health records and the clinical care and outcomes of patients with diabetes mellitus Ann Intern Med 2012 157 07 482 489 23027319 10.7326/0003-4819-157-7-201210020-00004 PMC3603566

[9] Conselho Federal de Medicina [Internet] Resolução CFM n. 1638 de 9 de agosto de 2002. Define prontuário médico e torna obrigatória a criação da Comissão de Revisão de Prontuários nas instituições de saúde2002 (cited 2013 Apr 26). Available at:http://www.portalmedico.org.br/resolucoes/cfm/2002/1638_2002.htm

[10] Nguyen L Bellucci E Nguyen L T Electronic health records implementation: an evaluation of information system impact and contingency factors Int J Med Inform 2014 83 11 779 796 25085286 10.1016/j.ijmedinf.2014.06.011

[11] HITEC Investigators Ancker J S Kern L M Edwards A Associations between healthcare quality and use of electronic health record functions in ambulatory care J Am Med Inform Assoc 2015 22 04 864 871 25896648 10.1093/jamia/ocv030 PMC11737649

[12] Kern L M Edwards A M Pichardo M Kaushal R Electronic health records and health care quality over time in a federally qualified health center J Am Med Inform Assoc 2015 22 02 453 458 25755124 10.1093/jamia/ocu049 PMC11737104

[13] Eggleston E M Klompas M Rational use of electronic health records for diabetes population management Curr Diab Rep 2014 14 04 479 24615333 10.1007/s11892-014-0479-z

[14] Corser W Yuan S Mixed influence of electronic health record implementation on diabetes order patterns for Michigan Medicaid AdultsJ Diabetes Sci Tech2015 10.1177/1932296815601689 PMC4773952 26292961

[15] Reed M Huang J Brand R Implementation of an outpatient electronic health record and emergency department visits, hospitalizations, and office visits among patients with diabetes JAMA 2013 310 10 1060 1065 24026601 10.1001/jama.2013.276733 PMC4503235

[16] Jolles D R Brown W W III King K B Electronic health records and perinatal quality: a call to midwives J Midwifery Womens Health 2012 57 04 315 320 22758354 10.1111/j.1542-2011.2012.00185.x

[17] Raglan G B Margolis B Paulus R A Schulkin J Electronic health record adoption among obstetrician/gynecologists in the United States: physician practices and satisfactionJ Healthc Qual 2015; [Epub ahead of print] 10.1111/jhq.12072 28481842

[18] EHR4CR WP7 Doods J Botteri F Dugas M Fritz F A European inventory of common electronic health record data elements for clinical trial feasibility Trials 2014 15 18 24410735 10.1186/1745-6215-15-18 PMC3895709

[19] Burke H B Sessums L L Hoang A Electronic health records improve clinical note quality J Am Med Inform Assoc 2015 22 01 199 205 25342178 10.1136/amiajnl-2014-002726 PMC4433367

[20] Kurtzman J H Wasserman E B Suter B J Glantz J C Dozier A M Measuring adequacy of prenatal care: does missing visit information matter? Birth 2014 41 03 254 261 24750400 10.1111/birt.12110

[21] National Academy of Science Institute of Medicine. Key capabilities of an electronic health record system: letter report Washington (DC) National Academies Press 2003 25057672

[22] Blumenthal D Glaser J P Information technology comes to medicine N Engl J Med 2007 356 24 2527 2534 17568035 10.1056/NEJMhpr066212

